# The mitigative effect of lotus root (*Nelumbo nucifera Gaertn*) extract on acute alcoholism through activation of alcohol catabolic enzyme, reduction of oxidative stress, and protection of liver function

**DOI:** 10.3389/fnut.2022.1111283

**Published:** 2023-01-11

**Authors:** Zihan Yang, Yuan Gao, Weijie Wu, Honglei Mu, Ruiling Liu, Xiangjun Fang, Haiyan Gao, Hangjun Chen

**Affiliations:** ^1^Food Science Institute, Zhejiang Academy of Agricultural Sciences, Hangzhou, China; ^2^Key Laboratory of Postharvest Handling of Fruits, Ministry of Agriculture and Rural Affairs, Hangzhou, China; ^3^Key Laboratory of Postharvest Preservation and Processing of Vegetables (Co-Construction by Ministry and Province), Ministry of Agriculture and Rural Affairs, Hangzhou, China; ^4^Key Laboratory of Fruits and Vegetables Postharvest and Processing Technology Research of Zhejiang Province, Hangzhou, China; ^5^Key Laboratory of Postharvest Preservation and Processing of Fruits and Vegetables, China National Light Industry, Hangzhou, China

**Keywords:** lotus root, acute alcoholism, antioxidant activity, active substance, acetaldehyde dehydrogenase

## Abstract

**Objectives:**

Lotus root (*Nelumbo nucifera Gaertn*) is a common medicinal-food dual-use vegetable. In this study, the effects of lotus root extract on acute alcoholism were investigated.

**Methods:**

The Walle-Hoch method was used to determine the ADH activity of lotus root extracts *in vitro*. Lotus root methanol extract were identified by UPLC-QTOF-MS/MS based metabolomics analysis. Then 109 active ingredients with achievable oral doses and drug-like properties were explored using the TCMSP platform. SwissTargetPrediction Database to predict lotus root treatment targets for acute alcoholismSTRING database (https://www.string-db.org/) was used to construct protein-protein interaction network graphs. Gene ontology (GO) functional, Kyoto Encyclopedia of Genes and Genomes (KEGG) pathway enrichment analysis of genes common to lotus root and alcoholism by Metascap database. Molecular docking simulations were performed using AutoDock 1.5.6 software. Animal experiments verified the relieving effect of lotus root extract on acute alcoholism after intervention.

**Results:**

Results indicated the methanol extract of lotus root showed the highest activation rate of ethanol dehydrogenase *in vitro* (18.87%). The 433 compounds of lotus root methanol extract were identified by UPLC-QTOF-MS/MS based metabolomics analysis. Bioinformatics analysis indicate that there were 224 intersectioning targets between lotus root extract and acute alcoholism. KEGG enrichment analysised shows that lotus root extract may play a role in treating acute alcoholism by intervening with the neuroactive ligand-receptor interaction pathway. The protein-protein interaction network (PPI) analysis found that HSP90AA1, MAPK1 and STAT3 played a key role in lotus root extract-modulated PPI networks. Molecular docking showed that (7R, 8S)-dihydrodihydrodipine cypressol had the best binding ability with MAPK1. Experiments in mice indicate that lotus root extract improved the activity of liver alcohol dehydrogenase (ADH), acetaldehyde dehydrogenase (ALDH), catalase (CAT), superoxide dismutase (SOD) and glutathione peroxidase (GSH-PX), increase glutathione (GSH) and reduce malondialdehyde (MDA) levels, decrease glutamate transaminase (AST), alanine transaminase (ALT) and alkaline phosphatase (AKP) in the serum of mice with acute alcoholism, and accelerate the metabolic rate of alcohol after drinking. This study reveals the mechanism of lotus root to alleviate acute alcoholism, which provides a basis for further research on functional foods using lotus root and offers new possibilities for the treatment of acute alcoholism.

**Conclusions:**

The results of the current study showed that the methanolic extract of lotus root had the highest activation rate of ethanol dehydrogenase. Network pharmacology results suggest that lotus root extract may play a role in the treatment of alcoholism by regulating signaling pathways, such as neuroactive ligand-receptor interactions, as well as biological processes, such as regulation of secretion, regulation of ion transport, response to lipopolysaccharides, and response to alcohol. Animal experiments confirmed the therapeutic effect of lotus root on acute alcoholism mechanistically through activation of alcohol catabolic enzyme, reduction of oxidative stress and protection of liver function.

## Introduction

With the increase in alcohol consumption worldwide, alcohol has been recognized as a cause of many liver diseases. The International Institute for Humanitarian Environment has listed alcohol consumption as one of the most essential factors endangering human health (1). Acute alcoholism usually refers to a series of body damage caused by a one-time overdose of alcohol, which mainly results in symptoms such as ataxia, respiratory depression and coma (2). Presently, the prevention and treatment of acute alcoholism, such as naloxone, metadoxine, and other drugs, can accelerate the metabolic rate of alcohol in the body to achieve the effect of alcohol detoxification. Although these drugs have certain effects in liver protection, most synthetic chemical drugs have toxic side effects. Therefore, it is essential to develop food-derived natural products with low toxic side effects because they target multiple molecules and pathways.

Lotus root (*Nelumbo nucifera Gaertn*) is China's largest and most productive aquatic vegetable. Lotus root mainly contains polyphenols, triterpenes, flavonoids, steroid compounds, and other active substances (3). According to modern pharmacological research, lotus root and its extracts have immunomodulatory, hypolipidemia, hypotensive, hypoglycemic, antioxidant, antibacterial, and other activities (4, 5). According to the ancient text, “Materia Medica,” it is known that the lotus root can detoxify alcohol and protect the liver. However, the specific detoxifying components in lotus roots are still unknown, and the synergistic effects between the components and the targets of action are yet to be elucidated.

To further determine the active components of the lotus root extract and elucidate its hepatoprotective effect, we used Ultra Performance Liquid Chromatography (UPLC) and Tandem mass spectrometry (MS/MS) to analyze the main chemical components of the lotus root extract to provide more comprehensive information for its specific active components. In this paper, based on the analysis of the active ingredients of lotus root, we used the bioinformatics research method to establish the active ingredient-target-disease-pathway from the holistic and systematic interactions among chemical components, targets, and diseases by using the traditional Chinese medicine systems pharmacology database and analysis platform (TCMSP), PubChem database, GeneCard database, and other bioinformatics platforms. The network diagram of active ingredient-target-disease-pathway was established, and the main active ingredients and core targets were docked and validated by molecular docking technology. Finally, we elaborated on the lotus root for the mechanism of action of rescuing acute alcohol poisoning.

## Materials and methods

### Preparation of lotus root extract

Lotus roots were purchased from a local farmers' association organization in Hangzhou, Zhejiang province, China. Fresh lotus root samples were placed in a mortar and pestle and ground into powder form. Several lotus root samples were weighed to 20 g each, and distilled water, 20% ethanol, 40% ethanol, 60% ethanol, 80% ethanol, anhydrous ethanol, 20% methanol, 40% methanol, 60% methanol, 80% methanol, and methanol were added at a material-to-liquid ratio of 1:10 (g:mL), respectively. The extraction method was assisted by ultrasonic extraction with power of 800 W and extraction temperature of 50°C for 1 h. The solvent extracts of lotus roots with different volume fractions were obtained by filtration. The extract was concentrated with a rotary evaporator and dried into a lyophilized powder using a vacuum freeze dryer (LABCONCO, America) and stored at −20°C for backup.

### Effect of lotus root extract on ethanol dehydrogenase (ADH) *in vitro*

The Walle-Hoch method (6) was used to determine the ADH activity of lotus root extracts *in vitro*. In a test tube, 0.375 mL of sodium pyrophosphate buffer (pH = 8.8), 0.25 mL of 0.027 mol/L NAD^+^, 0.125 mL of 12% ethanol, and 0.025 mL of different concentrations of lotus root extract was added in turn, mixed well and then incubated for 5 min at 25°C in a sealed incubator. After that, 0.025 mL of 3 U /mL ADH solution was added to the test tube. After shaking well, the solution was measured using the absorbance A at 340 nm by a microplate reader (Tecan, Switzerland), and the absorbance value was read every 10 s for 10 min, and the data were recorded, while the blank group was replaced by 0.025 mL of the sample solution with the solvent of the extract, and the rest of the steps were unchanged.


(1) $$\text{E = }\frac{\left(\text{A}\times \text{0}.\text{8}\right)}{\left({\text{E}}_{\text{w}}\times \text{6}.\text{2}\right)}$$

(2) $$\text{ADH activation rate }(\%)\text{ = }\frac{{\text{E}}_{\text{1}}{\text{−E}}_{\text{0}}}{{\text{E}}_{\text{0}}}\times \text{100}\%$$

Where E was the ADH enzyme activity, E_1_ was the sample group enzyme activity, E_0_ was the blank group enzyme activity, A was the value added of absorbance at 340 nm per 1 min for the initial linear part of the reaction, E_w_ was the enzyme content in the enzyme solution, 0.8 was the total volume of the reaction solution, 6.2 was the (NADH molar extinction coefficient at 340 nm, and A was the activation rate of ADH).

The *in vitro* activation rate of ethanol dehydrogenase was measured by different solvents of lotus root extracts, and the best active extract was screened for subsequent experimental analysis.

### UPLC-Q-TOF–MS/MS analysis

After thawing the samples from the refrigerator at −80°C, mixed them with vortex for 10 s. Took 50 mg of the sample after mixing, and placed 2 mL of the centrifuge tube. 1,200 μL 80% methanol internal standard extract was added. Scroll for 3 min Centrifuge (12,000 r/min, 4°C) for 10 min. The supernatant was filtered with a microporous filter membrane (0.22 μL) and stored in a sample flask for UPLC-Q-TOF-MS/MS. The model number of UPLC and MS/MS is Nexera X2 (SHIMADZU), and Biosystems 4500 QTRAP (AppliedBiosystems) (7).

### Chromatographic and mass spectrometry conditions

Chromatographic conditions: The chromatographic column was an Agilent SB-C18 model (1.8 μm, 2.1 mm ^*^ 100 mm). The mobile phase A was ultrapure water (with 0.1% formic acid) and phase B was acetonitrile (with 0.1% formic acid). The elution gradient: 5% of phase B at 0.00 min, linearly increasing to 95% of phase B at 9.00 min and maintaining at 95% for 1 min, decreasing to 5% of phase B at 10.00–11.10 min and equilibrating at 5% until 14 min. The flow rate was maintained at 0.35 mL/min, the reaction column temperature was 40°C, and the injection volume was 4 μL.

### ESI-Q TRAP-MS/MS

ESI electrospray source was used to collect data in positive and negative ion modes. The ESI source operation parameters were as follows: an ion source, turbo spray; source temperature 550°C; ion spray voltage (IS) 5,500 V (positive ion mode)/-4,500 V (negative ion mode). Ion source gas I (GSI), gas II (GSII), and curtain gas (CUR) are set at 50, 60, and 25.0 psi, respectively. Monitoring of a specific set of MRM ion pairs at each stage based on metabolites eluted during each period.

### Collection and screening of chemical components of lotus root

The information on the constituents of lotus root extracts obtained in 2.2 was entered into the Chinese Medicine Systematic Pharmacology Database and Analysis Platform (TCMSP, https://www.tcmsp-e.com/) for screening, with settings of oral bioavailability (OB) ≥30% and drug-likeness (DL) ≥0.18 were used to screen the eligible compounds as the effective compounds in lotus root (8, 9).

### Prediction of active ingredient targets of lotus root

PubChem database (10) (https://pubchem.ncbi.nlm.nih.gov/) was used to obtain the Canonical SMILES name of the active compound of lotus root. The compound Canonical SMILES name was entered into SwissTargetPrediction database (11) (http://old.swisstargetprediction.ch/), and the species origin was restricted to humans to predict the possible targets of the compound. Cytoscape 3.8.2 software (12) was used to construct the “active ingredient-target” network, and the Network analysis plugin was further used to analyze the degree and betweenness of the network characteristics to screen the key active ingredients of lotus root.

### Screening of potential targets for the effects of lotus root on alcoholism

We used “alcoholism” and “acute alcoholism” as search terms and entered them into the Online Human Mendelian Genetic Database (13) (OMIM, https://omim.org/); GeneCards database (14) (https://www.genecards.org/); Drugbank database (15) (https://www.drugbank.com/); PharmGkb database (16) (https://www.pharmgkb.org/); DisGeNET database (17) (https://www.disgenet.org/) to obtain disease targets. The disease targets obtained from each database were merged, and duplicate targets were removed to obtain the targets related to alcoholism. The target names were converted to protein names by uniprot database (18) (https://www.uniprot.org/), and the species' origin was limited to humans. The disease targets obtained above were intersected with the active ingredient targets of lotus root obtained by screening in 2.3.2 to obtain the active targets of lotus root for the treatment of acute alcoholism.

### Protein-protein interaction (PPI) construction and analysis

STRING database (19) (https://www.string-db.org/) was used to construct protein-protein interaction network graphs. The targets of lotus root for the treatment of alcoholism were imported into the database. The scoring value was set at a high confidence level of 0.900 to obtain the interaction relationships among the targets. The results were saved in TSV format and imported into Cytoscape 3.8.0 software, and the “Network Analysis” function was used to analyze and map. The interaction network was obtained by removing duplicate and isolated edges, using node size and color settings to reflect the size of the degree of network connectivity and edge thickness settings to reflect the size of the binding fraction.

### Core target identification

The CytoNCA plugin in Cytoscape 3.8.0 software was used to filter the core targets in the PPI network. The node degree (DC), betweenness (BC), closeness (CC), network (NC), eigenvector (EC), and local average connectivity-based centrality was calculated. Eigenvector centrality (EC) and Local Average Connectivity-based method (LAC) is used to filter the important targets in the interacting topological networks.

### Gene ontology (GO) and KEGG analysis

Gene ontology (GO) functional, Kyoto Encyclopedia of Genes and Genomes (KEGG) pathway enrichment analysis of genes common to lotus root and alcoholism by Metascap database (20) (https://metascape.org), GO enrichment analysis including biological process (BP), molecular function (MF), and cellular composition (CC). *P* < 0.05 was considered statistically significant, and a minimum number of enriched genes of 5 and enrichment factor >1.5 were used as screening criteria. The top20 GO functional categories and significantly enriched KEGG signaling pathways were selected. The network diagram of the lotus root component-target-pathway was constructed with Cytoscape 3.8.0 software.

### Molecular docking validation

In this study, molecular docking simulations were performed using AutoDock 1.5.6 software (21). Firstly, the structure files of the core targets were downloaded from the Protein Structure Database (22) (https://www1.rcsb.org/) and saved as PDB files. The core components of lotus root were downloaded as SDF files of the 3D structures of small molecules in the PubChem database. And the downloaded small molecules were energy minimized using Chem3D software and saved as mol2 files. Before docking, polar hydrogen atoms were added to the target protein using PyMOL software (23), and water molecules and ligands in the protein were removed. The docking mode was chosen as semi-flexible docking, the number of genetic algorithm calculations was set to 10, the number of populations was set to 150, and the maximum number of iterations was set to 2,500,000. the Lamarckian genetic algorithm (Lamrckian GA 4.2) was chosen as the docking algorithm, and the docking results were saved in pdbqt format. The docking results were saved in pdbqt format. The docking results were visualized by PyMOL software, and the “protein-molecule” docking interaction pattern was plotted.

### Animal experiment

The current study was conducted following the International Guiding Principles for Biomedical Research Involving Animals, approved by the Animal Ethics Committee of Zhejiang Academy of Agricultural Sciences (Committee approval #2021ZAASLA60). The animal experiments complied with the ARRIVE guidelines and were carried out in accordance with the U.K. Animals (Scientific Procedures) Act, 1986 and associated guidelines, EU Directive 2010/63/EU for animal experiments.

After all the mice adapted to the controlled conditions for 1 week, mice were randomly divided into five groups of eight each. Groups were named and treated as follows: (1) control group; (2) alcohol model (Alcohol) group, 0.85% normal saline oral administration once daily; (3) high concentration lotus root extract group (HD), 200 mg lotus root extract per kg body weight oral administration once daily; (4) middle concentration lotus root extract group (MD), 100 mg lotus root extract per kg body weight oral administration once daily; (5) low concentration lotus root extract group (LD) group, 50 mg lotus root extract per kg body weight oral administration once daily. Before ethanol treatment, all groups were fed once a day for seven consecutive days. On the 8th day, mice in each group were gavaged for 30 min. The mice in all groups except the control group were orally administered 52% edible alcohol at a dose of 12 mL per kg body weight to establish an acute alcoholism model. At the same time, the control group was orally administered distilled water. After 60 min of alcohol gavage, all the mice were sacrificed under anesthetization with diethyl ether asphyxiation. The liver tissues and serum were harvested and used for visceral index testing.

The blood ethanol content of each group of mice were determined using the detection kit provided by Nanjing Jiancheng Bioengineering Institute (Nanjing, Jiangsu, China) following the manufacturer's instructions.

### Determination of serum and liver parameters in mice

Detection kits were used to determine alanine aminotransferase (ALT), aspartate aminotransferase (AST), and alkaline phosphatase (AKP) activity in mouse serum. The liver tissues were used for the determination of ethanol dehydrogenase (ADH) and acetaldehyde dehydrogenase (ALDH) by UV colorimetric method, superoxide dismutase (SOD) activity by WST-1 method, glutathione peroxidase (GSH-Px) activity by colorimetric method, catalase (CAT) activity by ammonium molybdate method and malondialdehyde (MDA) activity by TBA method. These detection kits were provided by Nanjing Jiancheng Bioengineering Institute (Nanjing, Jiangsu, China) following the manufacturer's instructions.

### Data statistics and analysis

The data were expressed as mean ± standard deviation. Data processing and mapping using Excel and Origin-2021 software. ANOVA statistical analysis was performed using SPSS 25.0 with a 95% confidence interval (*P* < 0.05).

## Results

### Screening of ethanol dehydrogenase activity by fractions of lotus root extracts

**Figure 1:**
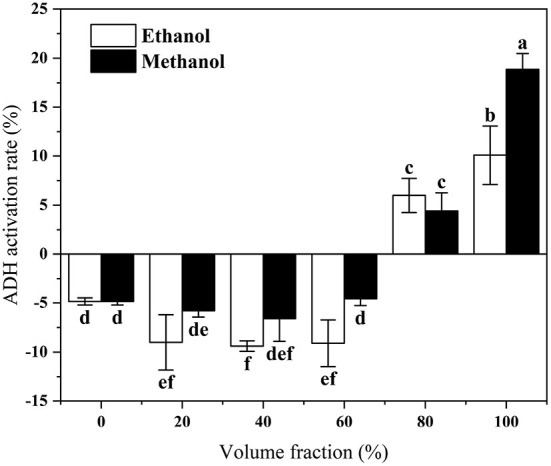
*In vitro* activation of ADH by lotus root extracts with different volume of fraction solvents. Different letters represent significant differences between groups.

In this experiment, different volume fractions of methanol and ethanol were used as solvents for the extraction of lotus roots. The ethanol dehydrogenase activity of each extract was determined using a modified Waller-Heho method. The activation rate of ethanol dehydrogenase by lotus root extracts with different volume fractions is shown in Figure 1. The activation rate of ethanol dehydrogenase by lotus root extract increased with increasing volume fraction of extraction solvent. The anhydrous methanolic extract of lotus root showed the most increased activation of ethanol dehydrogenase (18.87%). Therefore, this fraction was selected as the lotus root antidote fraction for the subsequent study.

### UPLC-QTOF-MS/MS analysis of lotus root extract

The total ion chromatograms (TICs) of the non-volatile components of the methanolic extract of lotus root are shown in Supplementary Figure 1. Supplementary Figure 1A is the TIC of the methanolic extract of the lotus root in positive ion mode, and Supplementary Figure 1B is the TIC of the methanolic extract of the lotus root in negative ion mode.

### Screening of active ingredients of lotus root extract

We analyzed lotus root methanol extract based on the above conditions of liquid chromatography and mass spectrometry. Four hundred and forty-three compounds were identified: 44 terpenoids, 72 alkaloids, 4 tannin, 43 lignan, 138 flavonoids, and 98 phenolic acids.

Screening of each compound is required to comply with the basic principles of network pharmacology. Using the TCMSP database, compounds that met the conditions of oral bioavailability (OB) ≥30% and drug-like properties (DL) ≥0.18 were used as candidates, yielding a total of 109 compounds, Supplementary Table 1 shows the mass spectra information of the top 30 compounds with relative contents after screening.

### Acute alcohol intoxication targets and lotus root treat target prediction

We obtained 51 relevant targets from the DisGeNET database, 778 alcohol-related targets were obtained from the GeneCards database, 6 relevant targets were obtained from the OMIM database, 49 relevant targets were obtained from the PharmGkb database and 16 alcoholism-related targets were obtained from the Drugbank database (Supplementary Figure 2A). By integrating the targets of each database, a total of 778 targets related to acute alcoholism were obtained. A total of 956 eligible targets of lotus root components were screened from the SwissTargetPrediction database, among which 224 targets related to acute alcoholism (Supplementary Figure 2B).

### Protein-protein interaction (PPI) analysis and core target screening

The PPI network reveals the potential connection between targets. To explore the possible mechanism of lotus root as a treatment against alcoholism, we imported the gene names of the 224 anti-alcoholism targets of lotus root to the STRING database to construct a PPI network. After hiding the unlinked protein nodes, we obtained 185 nodes and 592 edges (Supplementary Figure 3). Cytoscape 3.8.0 software was used to analyze the PPI network, and “Network Analysis” was used to analyze the network topology; the color and area of a node are related to the degree value; if the node has a larger degree value, it has a larger area and a darker color. According to the meso-number size, the larger the meso-number size, the thicker the line and the darker the color.

**Figure 2:**
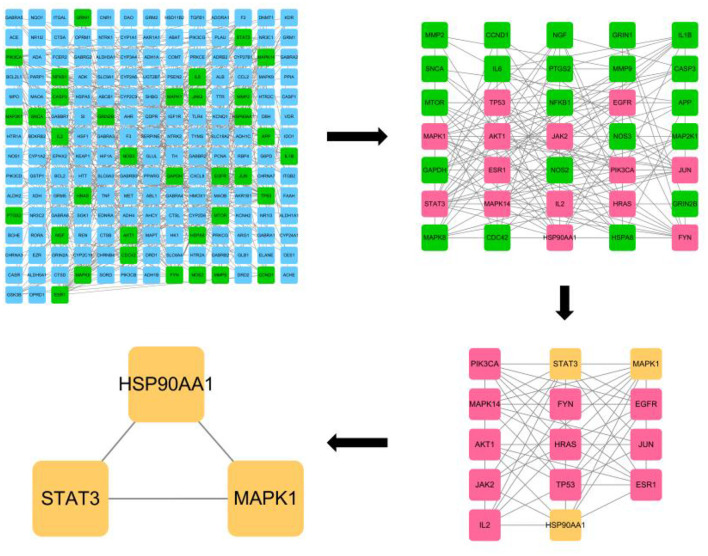
CytoNCA plugin based core target screening of lotus root for its effect on acute alcohol intoxication.

The CytoNCA plugin of Cytoscape 3.8.0 software was used to analyze the PPI network by calculating the Degree Centrality, Betweenness Centrality, Closeness Centrality, Network Centricity, and Local Average Connectivity-based method values of the network, respectively. Eigenvector Centrality and Local Average Connectivity-based method values were used to filter out the targets with each condition greater than the median value, and all targets were screened three times by this method (Figure 2). A total of 35 eligible targets with 168 edges were obtained after one screening. The second screening of these 35 genes yielded 14 core targets with 61 edges, and the third screening of these 14 genes yielded a total of three core targets with three edges. They were HSP90AA1, STAT3, and MAPK1, respectively.

### Gene ontology (GO) annotation analysis and KEGG pathway enrichment analysis results

From the GO enrichment analysis, 326 biological function entries were enriched at *p* < 0.05 (Supplementary Figure 4A). A total of 149 biological processes (BP) were involved, mainly including regulation of secretion, regulation of ion transport, response to lipopolysaccharide, response to alcohol, and regulation of hormone levels. The cellular component (CC) is enriched with 87 entries, mainly including postsynapse, dendrite, receptor complex, membrane raft, axon, postsynaptic specialization, vesicle lumen, and other components. Molecular functions (MF) were enriched to 90 entries, including neurotransmitter receptor activity, oxidoreductase activity, kinase binding, G protein-coupled amine receptor activity (G protein-coupled amine receptor activity, nuclear receptor activity, phosphotransferase activity, alcohol group as acceptor), lipid binding, catecholamine binding, and other molecular functions.

A total of 198 metabolic pathways were enriched by KEGG (Supplementary Figure 4B), and statistical analysis revealed that the co-expressed genes were mainly enriched in pathways related to Neuroactive ligand-receptor interaction (hsa04080), AGE-RAGE signaling pathway in diabetic complications, Pathways in cancer, Serotonergic synapse, cAMP signaling pathway in diabetic complications, cAMP signaling pathway in cancer, Signaling pathway in diabetic complications, Pathways in cancer, Serotonergic synapse, cAMP signaling pathway, etc. In addition to the KEGG-enriched top signaling pathway, alcoholism pathway was also enriched. Genes significantly enriched in the Neuroactive ligand-receptor interaction pathway (Supplementary Figure 4C) and alcoholism pathway (Supplementary Figure 4D) in KEGG Pathway analysis are marked in red.

### Construction of component target pathway network diagram

**Figure 3:**
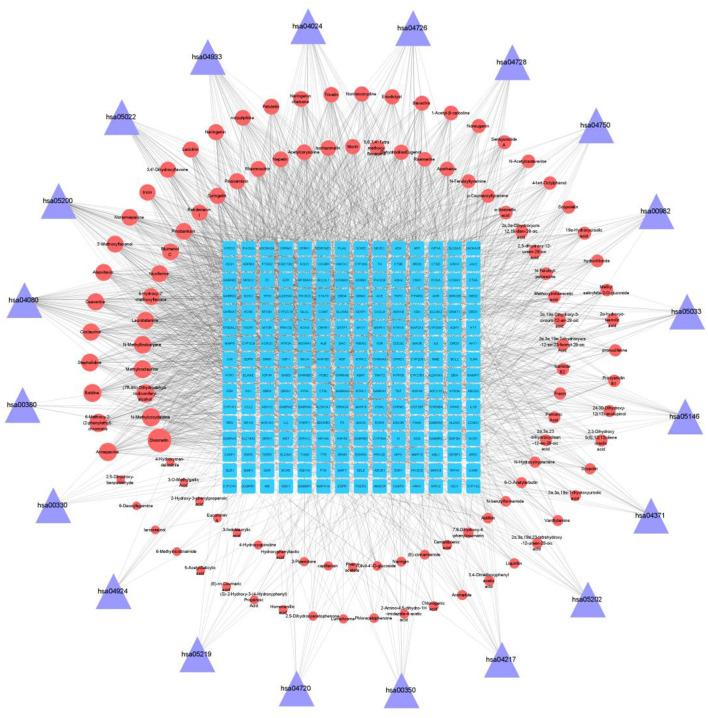
Compound-target-pathway network diagram.

Network diagram was constructed by combining the components of lotus root extract, acute alcoholism genes and the KEGG metabolic pathway obtained by enrichment (Figure 3). Blue squares represent the genes; purple triangles represent the KEGG pathway obtained by enrichment; red circles represent the substances contained in lotus root extract, and the ranking was based on the Degree value of each substance; the larger the degree value, the larger the area of red circles. The top seven compounds ranked according to the degree value were Diosmetin (degree = 58), 6-methoxy-2-(2-phenylethyl)chromone (degree = 38), (7R,8S)-Dihydrodehydrodiconiferyl alcohol (degree = 38), Armepavine (degree = 38), N-Methylcorydaldine (degree = 38), Boldine (degree = 37), Methylcoclaurine (degree = 37).

### Molecular docking validation of key components and core targets

**Figure 4:**
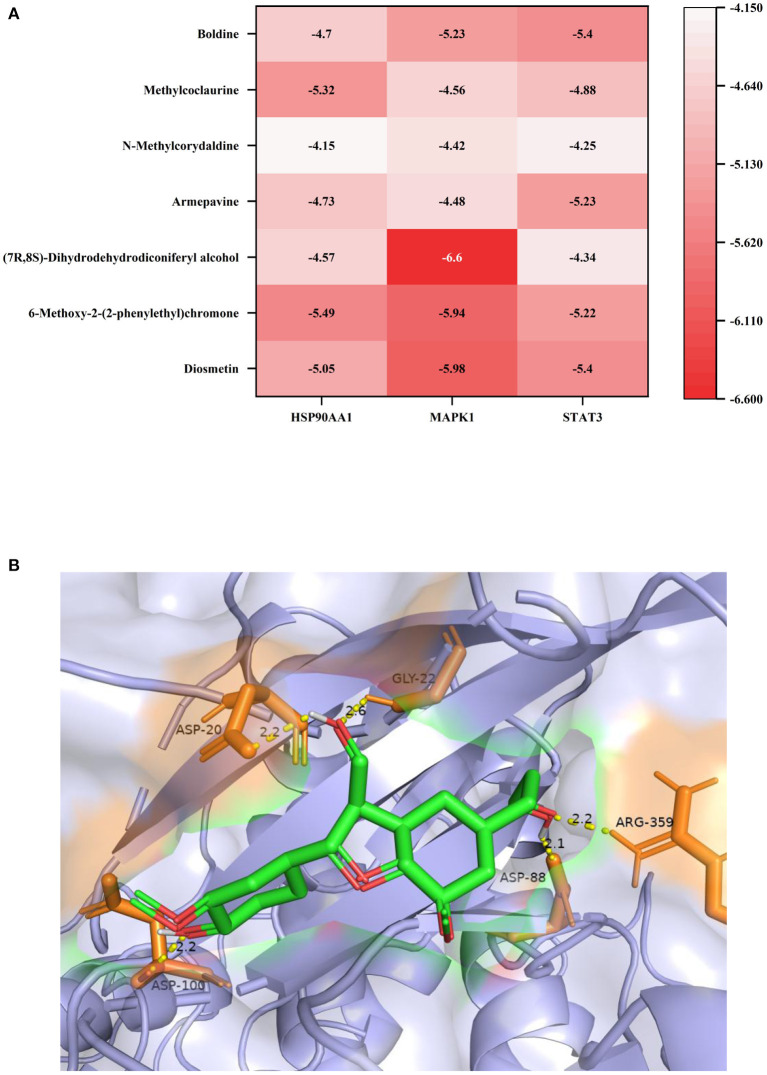
**(A)** Heat map of the binding energy of individual molecules to the receptor protein; **(B)** Docking results of (7R,8S)-Dihydrodehydrodiconiferyl alcohol with MAPK1 molecule.

Molecular docking verification of three key components of lotus root with seven core targets were analyzed using AutoDock software. Binding energy <0 kcal/mol means that the receptor and ligand can dock in the natural state, and binding energy <-1.2 kcal/mol means good docking results. Among the 21 docking results, (7R,8S)-Dihydrodehydrodiconiferyl alcohol has the lowest binding energy to MAPK1, which means that the formed protein-small molecule complexes are more stable (Figure 4). Among them, the hydroxymethyl at position 3 of (7R,8S)-Dihydrodehydrodiconiferyl alcohol molecule can form 2 hydrogen bonds with aspartic acid at position 20 and glycine at position 22 of MAPK1, 3-hydroxypropyl at position 5 can form 2 hydrogen bonds with arginine at position 359 and aspartic acid at position 88, and the phenolic hydroxyl group can form 1 hydrogen bond with aspartic acid at position 100.

### Effects of lotus root extract on ADH, ALDH, and CAT enzymes in mice

**Figure 5:**
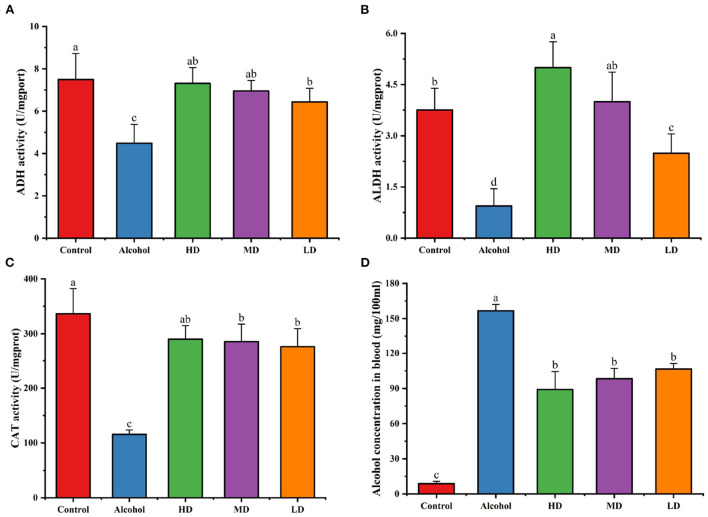
Effects of lotus root extract on hepatic ADH **(A)**, ALDH **(B)**, CAT **(C)** activities, and serum alcohol content in mice after gavage of alcohol **(D)**. Different letters represent significant differences between groups (N = 10, p < 0.05).

Ethanol dehydrogenase (ADH), acetaldehyde dehydrogenase (ALDH), and catalase (CAT) are essential enzymes in the metabolism of ethanol in the body, and the measurement of the activity of these enzymes in the liver can reflect the rate of alcohol metabolism to some extent. As Figure 5 shows, the vitality of ADH, ALDH, and CAT in the liver of the mice of alcohol-treated group was significantly decreased relative to that of the blank control group. After the intervention of lotus root extract, the vitality of ADH, ALDH, and CAT were significantly increased, among which, the lotus root extract activated ALDH, indicating that lotus root extract could accelerate the metabolism of ethanol and acetaldehyde in mice and reduce the damage caused by acute alcohol intoxication.

Figure 5D demonstrates the serum ethanol content of each group of mice 1 h after gavage after lotus root extract intervention. Compared with the control group, the blood alcohol concentration of mice in the alcohol-treated group was significantly higher. Compared with the control group, the blood alcohol concentration of mice in the experimental group treated with lotus root extract was significantly lower (*p* < 0.05). This result indicates that lotus root extract can significantly reduce the blood alcohol content in mice with acute alcohol intoxication.

### Effect of lotus root extract on liver indexes and antioxidant capacity in mice with acute alcohol intoxication

**Figure 6:**
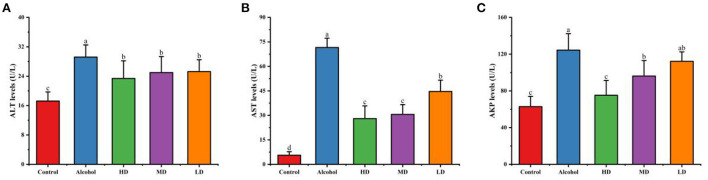
Effect of methanolic extract of lotus root on the levels of ALT **(A)**, AST **(B)**, and AKP **(C)** in the serum of mice with acute alcohol intoxication. Different letters represent significant differences between groups (N = 10, p < 0.05).

From Figure 6, the levels of AST, ALT, and AKP in the serum of mice in the acute alcohol intoxication group were significantly higher compared with the healthy control group (*p* < 0.05), indicating that the acute alcoholic liver injury in mice was successfully established; the high, medium, and low dose of methanolic extract of lotus root significantly decreased the activities of AST, ALT, and AKP in the serum compared to those on the control diet. The results showed that lotus root extract could reduce liver injury caused by alcohol in mice.

**Figure 7:**
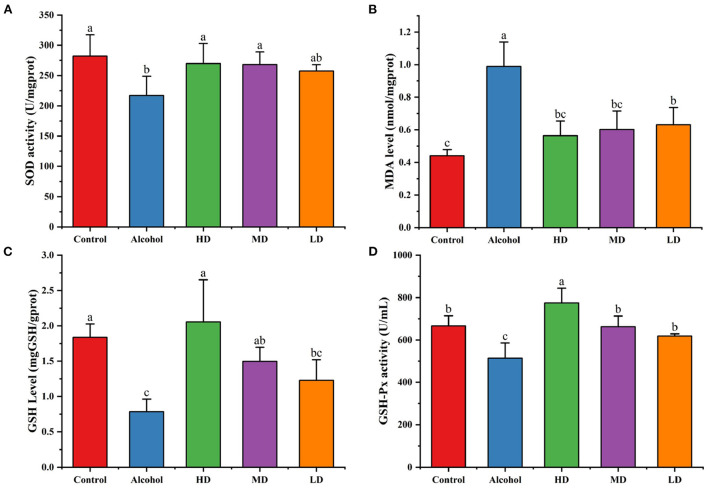
Addictive effects on hepatic SOD **(A)**, MDA **(B)**, and GSH **(C)** levels. **(D)** GSH-Px activity. Different letters represent significant differences between groups (N = 10, p < 0.05).

From Figure 7, compared with the healthy control group, the SOD activity and GSH level in the liver tissue of mice in the acute alcohol intoxication mice were decreased (*p* < 0.05) and the MDA level was increased (*p* < 0.05). Lotus root extracts increased the SOD activity and GSH-Px level and decreased the lipid peroxidation products caused by oxidative stress in the liver tissue homogenate of mice (*p* < 0.05). The levels of MDA decreased (*p* < 0.05), indicating that lotus root extract could enhance the antioxidant capacity of the liver of mice with acute alcoholic liver injury and it had a certain protective effect on acute alcoholic-caused liver injury.

## Discussion

In this paper, we screened ethanol dehydrogenase activation activity of the different lotus root extracts and concluded that the methanol extract of lotus root had the highest activation capacity for ethanol dehydrogenase. UPLC-QTOF-MS/MS can be used to obtain high-resolution and high-precision mass spectrometry data, and the relevant chemical components can be characterized more accurately according to the structures of the components and their bond-breaking patterns, which have been widely used for the identification of natural herbal ingredients. In this study, 109 potentially active compounds in the methanolic extract of lotus root were obtained by relevant screening conditions.

The targets of these components had a total of 224 intersecting targets with the targets of acute alcoholism. It is suggested that lotus root extract may help to alleviate the symptoms of acute alcoholism. The mechanism of action of lotus root in treating acute alcoholism was further investigated by enrichment analysis. The results indicated that the neuroactive ligand-receptor interaction pathway is likely to be the key pathway for treating acute alcoholism. The network diagram of the component-target-metabolism pathway was constructed to obtain the relationship between the three interactions, and the top seven components of lotus root with Degree value obtained were Diosmetin, 6-methoxy-2-(2-phenylethyl)chromone, (7R,8S)-Dihydrodehydrodiconiferyl alcohol, Armepavine, N-Methylcorydaldine, Boldine, Methylcoclaurine. These were mainly alkaloids and flavonoids, which suggested that alkaloids and flavonoids might be the main alcoholic and hepatoprotective components in lotus root. The intersecting targets were subjected to protein-protein interaction analysis, and these targets were screened in combination with the cytoNCA plugin of Cytoscape software. The three core targets were obtained were heat shock protein 90 (HSP90AA1), signal transducer and activator of transcription (STAT3), and mitogen-activated protein kinase 1 (MAPK1).

MAPK1 is an essential component of the MAP kinase/signal transduction pathway. It is a major member of the MAPK family, which plays an important role in cell growth, differentiation, inflammatory response, and other pathological processes. Inhibition of MAPK protein expression could effectively inhibit alcoholic liver injury (24). Our molecular docking results suggest that (7R,8S)-Dihydrodehydrodiconiferyl alcohol from lotus root had the most substantial binding capacity to MAPK1. The Signal transducer and activator of transcription 3 is an essential nuclear transcription factor, and studies have shown that STAT3 is significantly expressed in all human multi-inflammatory diseases (25). Further, STAT3 is significantly expressed in human multi-inflammatory diseases. Alcohol activates the IL-6/STAT3 pathway, which is a pathway that mediates the inflammatory response. When the body undergoes an inflammatory response, IL-6 is synthesized in large quantities in the liver and binds to its receptor, IL-6R, to form a complex, which then activates STAT3, in addition to activating the NF-κB signaling pathway, prompting the transfer of NF-κB into the nucleus and mediating the inflammatory response (26, 27). All the main active components of lotus root can bind to STAT3, and inhibit binding to key sites on STAT3 can suppress the alcohol-induced inflammatory response and reduce the risk of alcoholic hepatitis. Heat shock protein 90 (HSP90AA1) is a molecular chaperone protein that regulates cell survival, proliferation, and apoptosis by maintaining the structural and functional integrity of proteins critical for cell survival (28). Studies have shown that heat shock protein 90 inhibitors can induce apoptosis in hepatic stellate cells and inhibit stellate cell activation followed by induction of apoptosis. This finding implies that inhibition of heat shock protein 90 activity may be an effective therapeutic strategy for treating liver disease (28).

Finally, the protective effect of lotus root extract on acute alcohol intoxicated mice was investigated. Alcohol is mainly metabolized in the liver. After heavy alcohol consumption, the alcohol metabolite acetaldehyde and the reactive oxygen radicals generated during its metabolism can damage liver cells, resulting in a large depletion of endogenous SOD and GSH (29, 30). Reactive oxygen species can also interact with unsaturated fatty acids on cell membranes, leading to cell membrane permeability changes. AST, ALT, and AKP in liver cells will enter the bloodstream, manifesting an increase in these indicators. In addition, the membrane lipid peroxidation caused by alcohol consumption also generates a large amount of MDA that accumulates in the body. Lotus root extract can accelerate the metabolism of alcohol by activating the activity of ADH, ALDH, and CAT and reduce the level of ethanol in the serum after drinking to achieve the effect of rapid alcohol detoxification and sobriety. Meanwhile, the polyphenolic compounds abundant in lotus root have substantial free radical scavenging ability and exhibit good antioxidant capacity, which can significantly reduce the elevated serum AST, ALT, and AKP activities caused by alcohol consumption. Lotus root extract can also restore the reduced GSH depleted by alcohol metabolism, and the reduced GSH can improve liver detoxification capacity and repair damaged liver cells. Clinical studies have shown that reduced glutathione has certain therapeutic effects on patients with alcoholism (31).

## Conclusions

The results of the current study showed that the methanolic extract of lotus root had the highest activation rate of ethanol dehydrogenase. Network pharmacology results suggest that lotus root extract may play a role in the treatment of alcoholism by regulating signaling pathways, such as neuroactive ligand-receptor interactions, as well as biological processes, such as regulation of secretion, regulation of ion transport, response to lipopolysaccharides, and response to alcohol. Animal experiments confirmed the therapeutic effect of lotus root on acute alcoholism mechanistically through activation of alcohol catabolic enzyme, reduction of oxidative stress and protection of liver function.

## Data availability statement

The original contributions presented in the study are included in the article/Supplementary material, further inquiries can be directed to the corresponding authors.

## Ethics statement

The animal study was reviewed and approved by Animal Ethics Committee of Zhejiang Academy of Agricultural Sciences (Committee approval #2021ZAASLA60).

## Author contributions

ZY: methodology, validation, data curation, and writing—original draft. YG: conceptualization and writing—review and editing. WW, HM, RL, and XF: formal analysis and resources. HG: conceptualization and methodology. HC: conceptualization, project administration, data curation, and supervision. All authors contributed to the article and approved the submitted version.
