# Supplementary material for: The mitigative effect of lotus root (Nelumbo nucifera Gaertn) extract on acute alcoholism through activation of alcohol catabolic enzyme, reduction of oxidative stress, and protection of liver function
Source: Front Nutr. 2023 Jan 11;9:1111283. doi: 10.3389/fnut.2022.1111283 (PMC9875029; doi:10.3389/fnut.2022.1111283)
Supplement: Supplementary file 1 [file Data_Sheet_1.docx]

**Supplementary Materials**

Table S1. UPLC-Q-TOF-MS/MS information of the top 30 compounds with relative content in lotus root extracts screened by network pharmacology

| **Compounds** | **Formula** | **Q1 (Da)** | **Q3 (Da)** | **Molecular Weight (Da)** | **Rt (min)** | **fragments** | **Ionization model** | **Class I** | **Relative abundance** |
| --- | --- | --- | --- | --- | --- | --- | --- | --- | --- |
| 2,3-dihydroxy-12-ursen-28-oic acid | C30H48O4 | 471.34 | 471.34 | 472.36 | 9.06 | 471.35,469.89,453.35,407.33,425.34,409.35,411.33 | [M-H]- | Terpenoids | 18858000.00 |
| 3-Indoleacrylic acid | C11H9NO2 | 188.07 | 118.07 | 187.06 | 2.41 | 188.06,118.07,146.06,143.07,115.05,144.08,117.06 | [M+H]+ | Alkaloids | 8328000.00 |
| 2,5-Dihydroxybenzaldehyde | C7H6O3 | 137.02 | 93.03 | 138.03 | 3.32 | 137.05,93.04,65.04,92.75,75.02,91.02,67.02 | [M-H]- | Phenolic acids | 7816900.00 |
| 6-Deoxyfagomine | C6H13NO2 | 132.10 | 57.10 | 131.10 | 1.2 | 132.1,86.1,69.07,57.06,56.05,58.07,55.06 | [M+H]+ | Alkaloids | 6273600.00 |
| Caaverine | C17H17NO2 | 268.13 | 191.10 | 267.13 | 3.71 | 268.13,191.09,219.09,251.11,236.09,190.08,189.07 | [M+H]+ | Alkaloids | 5988100.00 |
| 2α,3α,23-trihydroxyolean-12-en-28-oic acid | C30H48O5 | 487.34 | 487.34 | 488.35 | 7.27 | 487.34,457.33,472.08,443.35,485.83,469.29,440.06 | [M-H]- | Terpenoids | 4287200.00 |
| N-Methylasimilobine | C18H19NO2 | 282.15 | 236.20 | 281.14 | 3.79 | 282.15,219.09,191.09,251.12,236.09,190.08,189.07 | [M+H]+ | Alkaloids | 3357700.00 |
| N-benzylformamide | C8H9NO | 136.07 | 91.05 | 135.07 | 1.44 | 136.07,91.05,119.04,65.04,107.05,92.02,77.04 | [M+H]+ | Alkaloids | 2524000.00 |
| Nuciferine | C19H21NO2 | 296.16 | 250.10 | 295.38 | 4.63 | 296.16,265.13,250.1,234.1,235.07,219.08,233.09 | [M+H]+ | Alkaloids | 2161600.00 |
| Pinocembrin | C15H12O4 | 255.07 | 151.00 | 256.07 | 7.03 | 255.07,213.06,151,171.05,145.07,211.08,107.01 | [M-H]- | Flavonoids | 2048500.00 |
| 4-Hydroxymandelonitrile | C8H7NO2 | 150.06 | 61.01 | 149.05 | 1.24 | 150.06,56.05,61.01,122.06,133.03,95.05,87.03 | [M+H]+ | Alkaloids | 1895300.00 |
| Scopoletin | C10H8O4 | 193.05 | 133.03 | 192.04 | 4.25 | 193.04,133.03,178.02,137.06,122.03,150.03,94.04 | [M+H]+ | Lignans and Coumarins | 985820.00 |
| Armepavine | C19H23NO3 | 314.17 | 283.14 | 313.39 | 3.55 | 314.17,283.14,107.05,189.09,252.11,268.11,145.06 | [M+H]+ | Alkaloids | 967160.00 |
| Procyanidin B1* | C30H26O12 | 577.14 | 425.10 | 578.14 | 2.66 | 577.14,407.08,289.07,425.09,451.11,125.02,287.06 | [M-H]- | Tannins | 903080.00 |
| Methoxyindoleacetic acid | C11H11NO3 | 206.10 | 147.50 | 205.07 | 2.48 | 206.1,147.5 | [M+H]+ | Alkaloids | 711630.00 |
| Tyramine | C8H12ClNO | 138.09 | 77.04 | 173.64 | 0.83 | 138.09,77.04,121.06,103.05,91.05,93.07,51.02 | [M+H]+ | Alkaloids | 619570.00 |
| Methylcoclaurine | C18H21NO3 | 300.15 | 269.12 | 299.15 | 2.96 | 300.15,269.12,107.05,237.09,175.07,257.12,197.1 | [M+H]+ | Alkaloids | 569000.00 |
| N-Hydroxytryptamine | C10H12N2O | 177.10 | 160.30 | 176.10 | 1.55 | 177.09,160.08,132.08,115.05,117.06,142.07,159.07 | [M+H]+ | Alkaloids | 505800.00 |
| 2-Piperidone | C5H9NO | 100.08 | 56.05 | 99.13 | 2.11 | 100.08,56.05,55.05,82.07,72.08,57.03,54.03 | [M+H]+ | Alkaloids | 479770.00 |
| Roemerine | C18H17NO2 | 280.13 | 249.08 | 279.13 | 4.39 | 280.13,249.1,191.09,219.08,201.07,203.09,190.08 | [M+H]+ | Alkaloids | 474120.00 |
| p-Coumaroyltyramine | C17H17NO3 | 284.13 | 147.04 | 283.12 | 4.85 | 284.13,147.05,121.07,119.05,91.06,93.07,103.06 | [M+H]+ | Alkaloids | 473490.00 |
| Aporheine | C18H17NO2 | 280.13 | 249.10 | 279.30 | 4.68 | 280.13,249.1,219.08,191.09,249.12,201.07,280.13 | [M+H]+ | Alkaloids | 374400.00 |
| (E)-m-Coumaric acid | C9H8O3 | 163.04 | 119.05 | 164.16 | 4.23 | 163.04,119.05,91.06,93.04,117.04,65.04,89.04 | [M-H]- | Phenolic acids | 293870.00 |
| Phenyl acetate | C8H8O2 | 135.05 | 92.03 | 136.05 | 4.2 | 135.05,59.01,61.99,65.05,67.02,70,92.03 | [M-H]- | Phenolic acids | 259150.00 |
| Hydroxyphenyllactic acid | C9H10O4 | 181.05 | 135.04 | 182.06 | 2.45 | 181.05,135.04,119.05,163.04,134.04,72.99,181.05 | [M-H]- | Phenolic acids | 225140.00 |
| tricin | C17H14O7 | 331.08 | 315.05 | 330.07 | 5.75 | 331.08,315.05,270.05,287.05,286.05,301.03,242.06 | [M+H]+ | Flavonoids | 213390.00 |
| Norarmepavine | C18H21NO3 | 300.15 | 283.13 | 299.15 | 3.43 | 300.15,283.14,107.05,189.09,252.11,268.11,145.06 | [M+H]+ | Alkaloids | 186380.00 |
| Pinobanksin | C15H12O5 | 271.06 | 151.00 | 272.07 | 5.45 | 271.06,151.01,119.05,107.01,177.02,93.04,65 | [M-H]- | Flavonoids | 183090.00 |
| Naringenin | C15H12O5 | 271.06 | 151.00 | 272.07 | 5.54 | 271.06,151,119.05,271.06,107.01,177.02,65 | [M-H]- | Flavonoids | 178780.00 |
| Eriodictyol | C15H12O6 | 287.06 | 135.00 | 288.06 | 5.03 | 287.06,135.05,151.01,107.01,65,134.04,83.01 | [M-H]- | Flavonoids | 153330.00 |

Q1 is the parent ion molecular weight, Q3 is the daughter ion molecular weight in Da

| **A**  **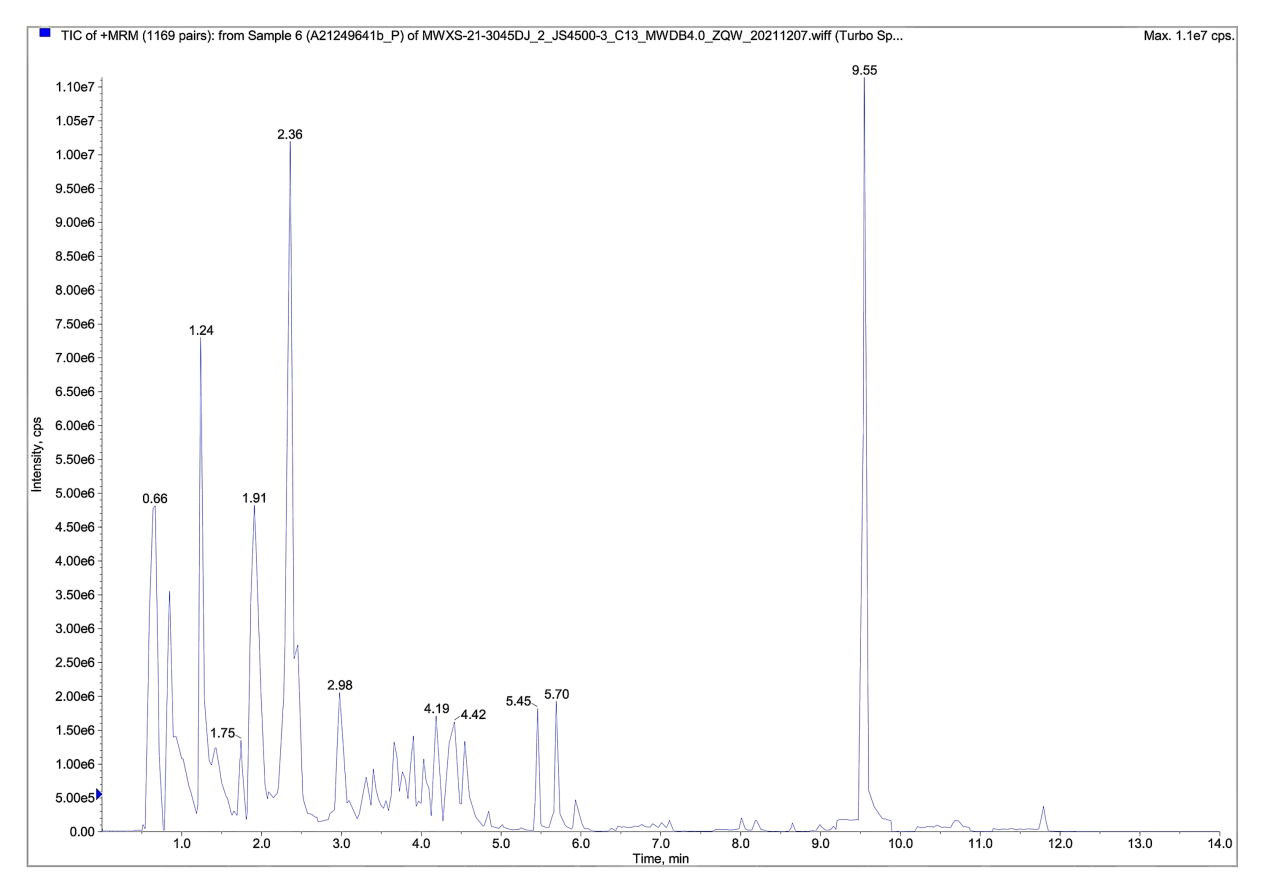** |
| --- |
| **B**  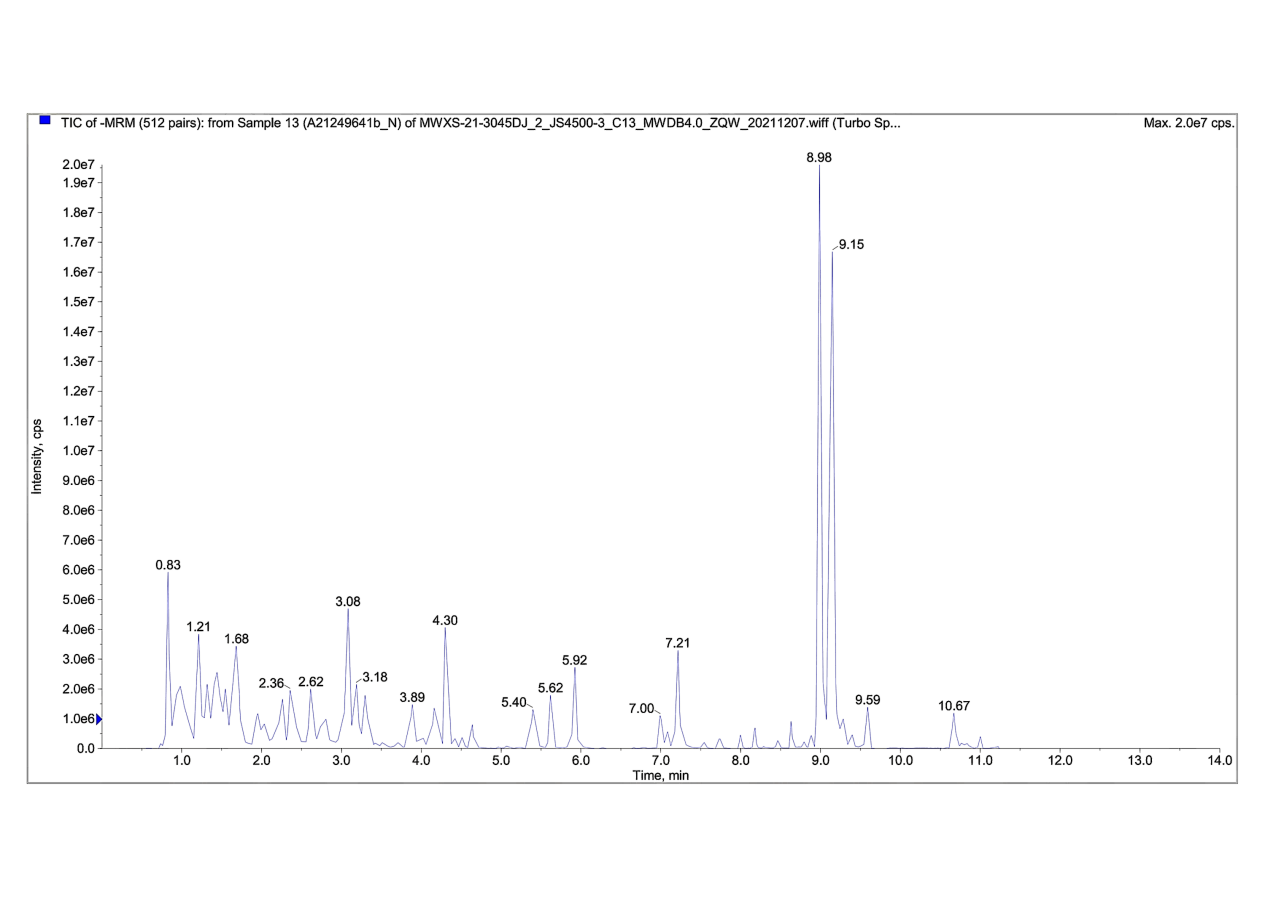 |

**Fig. S1** Total ion chromatograms (TICs) of lotus root extracts determined by ultra performance liquid chromatography-quadrupole-time of flight tandem mass spectrometry (UPLC/Q-TOF-MS/MS). a: TICs in positive ion mode; b: TICs in negative ion mode


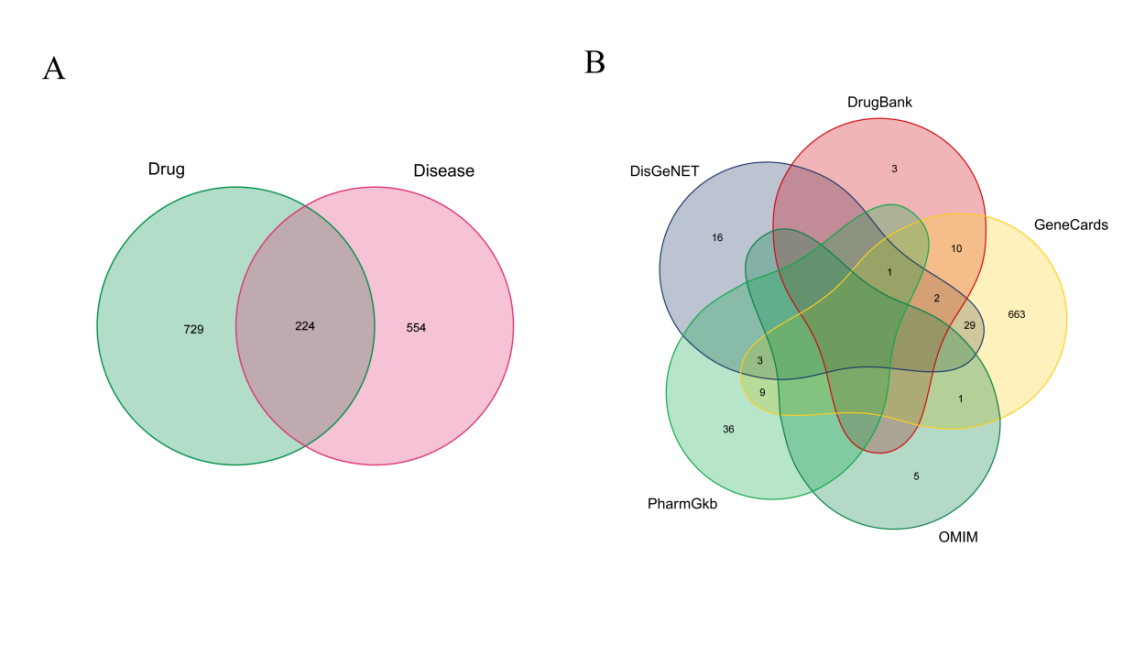


**Fig. S2** Venn diagram of constituent targets of lotus root extract and acute alcoholism-related targets (A).Venn diagram of acute alcoholism-related targets of five disease databases (B).


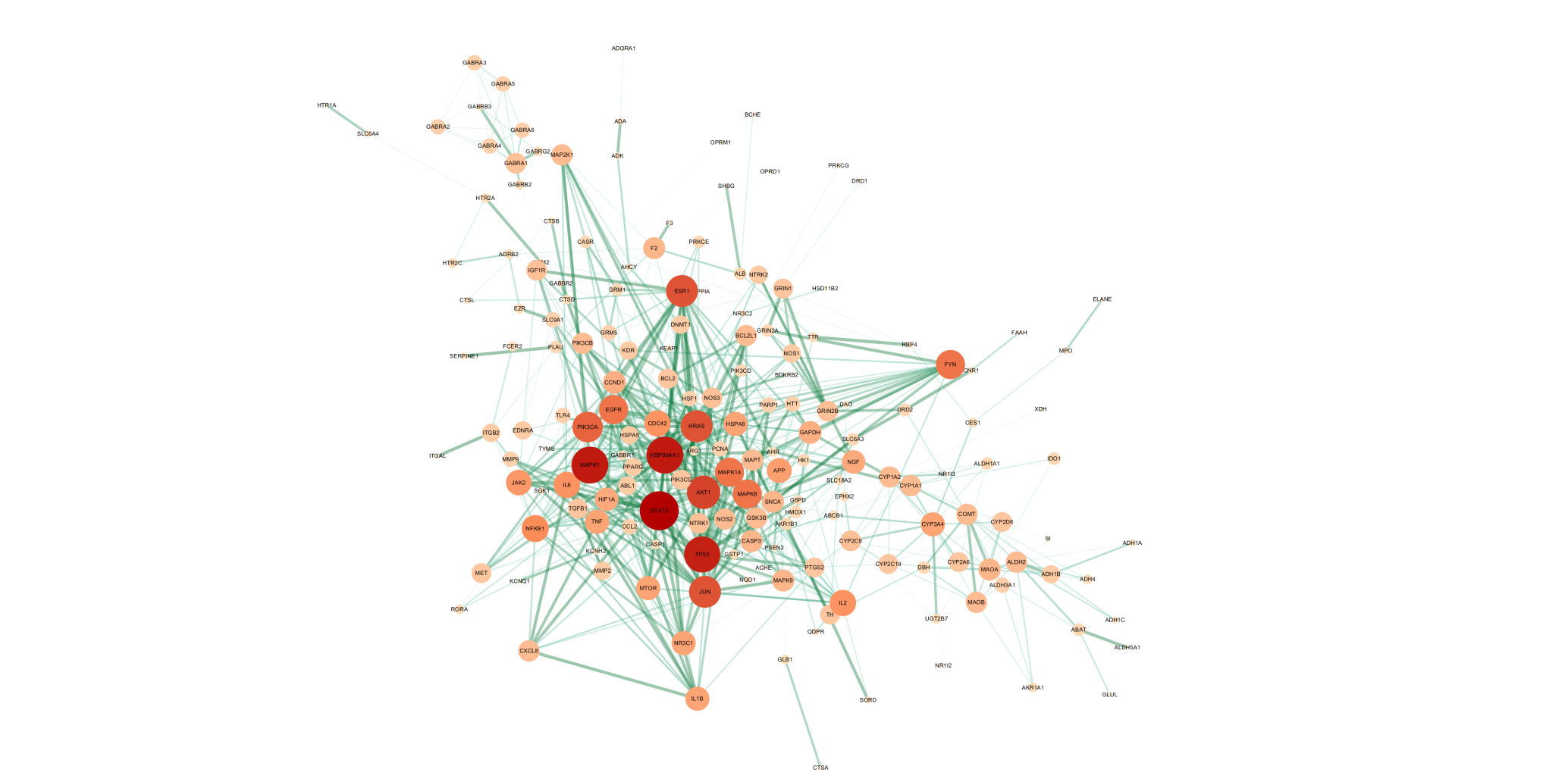


**Fig. S3** Protein-protein interactions


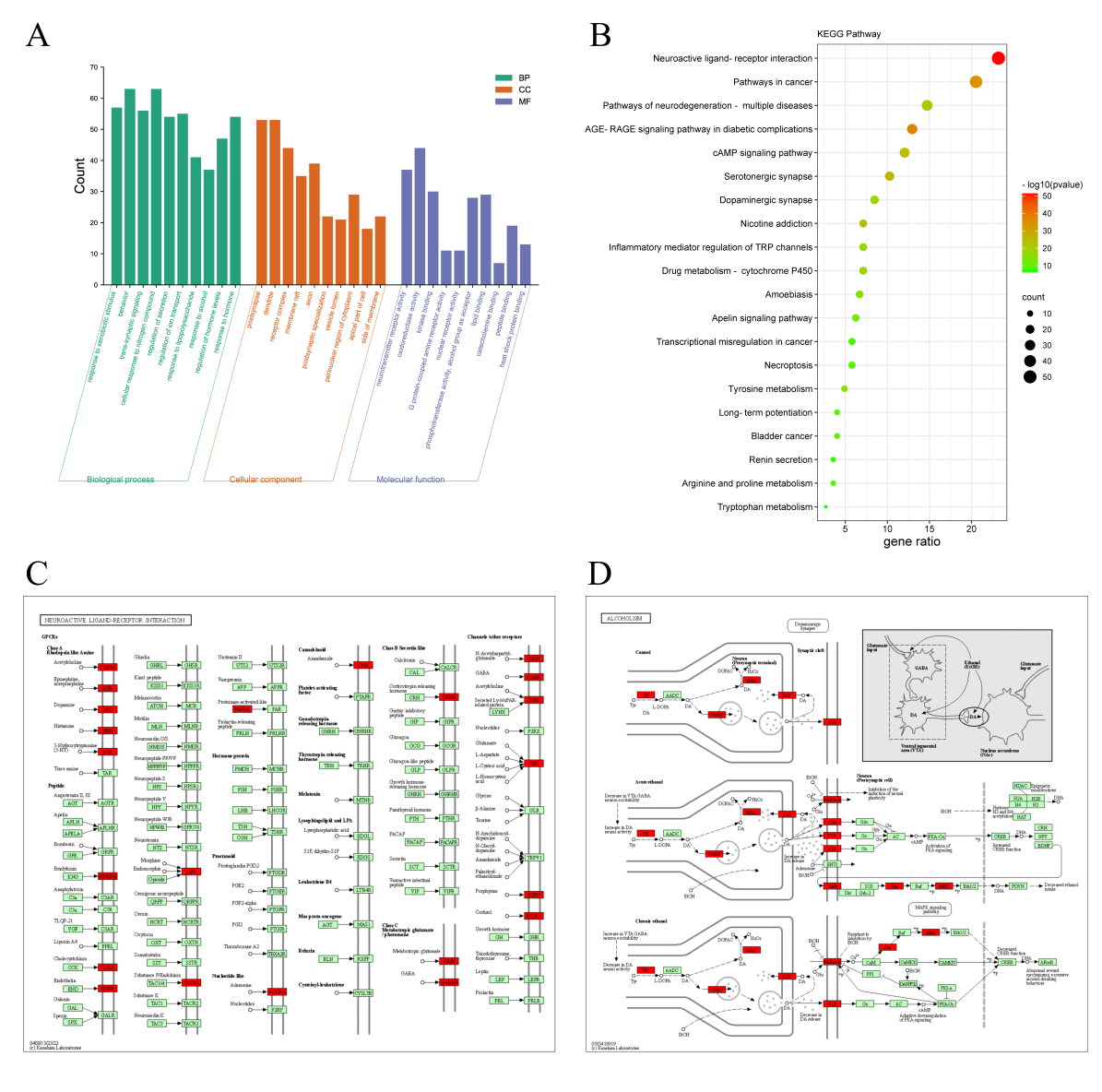


**Fig. S4** GO enrichment analysis (A) and KEGG pathway enrichment analysis (B), Neuroactive ligand-receptor interaction pathway (C), and alcoholism pathway (D).
